# Metal-Free ALS Variants of Dimeric Human Cu,Zn-Superoxide Dismutase Have Enhanced Populations of Monomeric Species

**DOI:** 10.1371/journal.pone.0010064

**Published:** 2010-04-09

**Authors:** Anna-Karin E. Svensson, Osman Bilsel, Can Kayatekin, Jessica A. Adefusika, Jill A. Zitzewitz, C. Robert Matthews

**Affiliations:** Department of Biochemistry and Molecular Pharmacology, University of Massachusetts Medical School, Worcester, Massachusetts, United States of America; Griffith University, Australia

## Abstract

Amino acid replacements at dozens of positions in the dimeric protein human, Cu,Zn superoxide dismutase (SOD1) can cause amyotrophic lateral sclerosis (ALS). Although it has long been hypothesized that these mutations might enhance the populations of marginally-stable aggregation-prone species responsible for cellular toxicity, there has been little quantitative evidence to support this notion. Perturbations of the folding free energy landscapes of metal-free versions of five ALS-inducing variants, A4V, L38V, G93A, L106V and S134N SOD1, were determined with a global analysis of kinetic and thermodynamic folding data for dimeric and stable monomeric versions of these variants. Utilizing this global analysis approach, the perturbations on the global stability in response to mutation can be partitioned between the monomer folding and association steps, and the effects of mutation on the populations of the folded and unfolded monomeric states can be determined. The 2- to 10-fold increase in the population of the folded monomeric state for A4V, L38V and L106V and the 80- to 480-fold increase in the population of the unfolded monomeric states for all but S134N would dramatically increase their propensity for aggregation through high-order nucleation reactions. The wild-type-like populations of these states for the metal-binding region S134N variant suggest that even wild-type SOD1 may also be prone to aggregation in the absence of metals.

## Introduction

**Figure 1:**
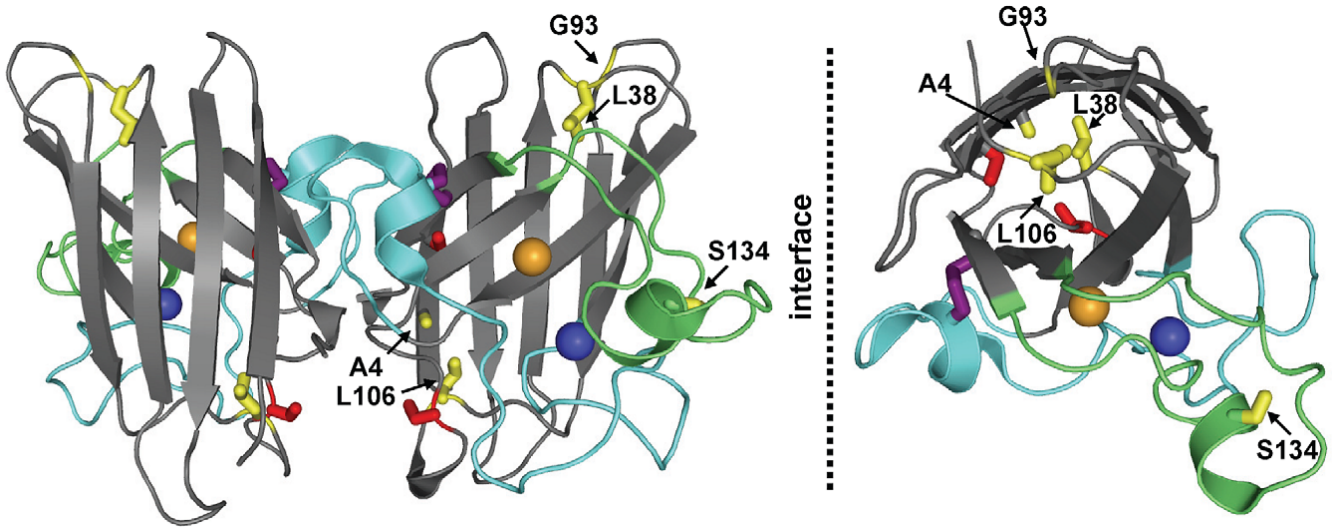
Ribbon diagram of human Cu,Zn superoxide dismutase. (left) The free cysteine residues at the sites of the C6A and C111S mutations and the C57–C146 disulfide bond are shown in red and purple ball-and-stick models, respectively. The Zn-binding loop (loop IV; residues 49–82) is shown in pale blue, and the electrostatic loop (loop VII; residues 121–142) is in pale green. The Zn^2+^ and Cu^2+^ ions are shown as blue and orange spheres, respectively. The sites of ALS mutations (A4V, L38V, G93A, L106V, and S134N) are highlighted in yellow ball-and-stick models. (right) One monomer of SOD1 turned 90° around a horizontal axis toward the viewer. The figure was generated with PyMOL [52] using pdb code 2c9v [53].

Amyotrophic lateral sclerosis is a devastating neurodegenerative disease that affects 2 in every 100,000 people worldwide [1]. Approximately 10% of all ALS cases are inherited, i.e., familial (fALS), of which 20% are caused by mutations in the *SOD1* gene that codes for the cytosolic enzyme Cu,Zn superoxide dismutase (SOD1). SOD1 is a homo-dimeric protein, whose 153-residue subunits fold into a β-barrel composed of eight anti-parallel β-strands arranged in a Greek key motif [2]; short stretches of helix form parts of the subunit interface and electrostatic loop (Figure 1). The β-barrel structure of SOD1 provides the scaffold for the electrostatic and Zn-binding loops [3]. Copper enables the redox cycle responsible for the dismutation of superoxide anion to molecular oxygen and hydrogen peroxide [4], [5], and zinc stabilizes the native dimeric conformation [6], [7]. An intra-molecular disulfide bond between Cys57 and Cys146 covalently links the zinc-binding loop with the C-terminal β-strand, β8, and stabilizes the native dimeric structure [8]–[10].

Over 140 point mutations dispersed throughout the sequence of SOD1 (http://alsod.iop.kcl.ac.uk/Als/) can cause ALS by exerting a gain-of-function toxicity [11]. Although a variety of mechanisms for this toxicity have been proposed [1], the appearance of SOD1-containing aggregates in neurons of patients afflicted with ALS [12], [13] is consistent with a possible role for protein misfolding and aggregation in disease.

Controversy currently exists as to whether aggregation-prone monomeric species, small oligomers or macroscopic aggregates are the cytotoxic species [12], [13]. Support for a crucial role for monomeric SOD1 in aggregation is provided by the results of studies in which SOD1 aggregates found in spinal cords extracted from a human A4V SOD1 patient [14] and from various ALS mouse models [14], [15] reacted with antibodies specific for the monomeric state [14]. Reduced monomeric apo-SOD1 has also been implicated to initiate aggregation of dimeric holo-SOD1 at neutral pH and 37°C [16], and disulfide-reduced SOD1 species are enriched in the spinal cords of ALS mice [17]. Whichever species proves to be the toxic agent, monomers of limited solubility would likely play a central role in pathogenesis, either directly or as the precursor to small oligomers or high-molecular-weight aggregates.

The misfolding and aggregation hypothesis has motivated a variety of biophysical studies of the effects of the mutations on the stability and folding mechanism of SOD1 [7], [18]–[23]. All studies conclude that a three-state mechanism, 2U
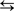
2M
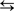
N_2_, provides an accurate description of the equilibrium mechanism for the metal-free (apo) system. The U and N_2_ states represent the unfolded monomeric and native dimeric forms of SOD1,respectively, and M represents the folded monomeric form. In the presence of zinc and/or copper, the same mechanism is operative. The N_2_ and M states are both capable of binding metals at room temperature, neutral pH and in the absence of denaturational stress. By contrast, the U state has a greatly reduced affinity for both metals under these conditions [6], [7], [24], [25].

If the aggregation of the monomeric M or U species is involved with toxicity in fALS, it might be expected that ALS-inducing variants would enhance its population. With the exception of one thermodynamic study that required the addition of stabilizing agents to measure stabilities [22], previous folding studies of ALS variants have not directly calculated populations. The goal of the present study is to use a global kinetic analysis to quantitatively assess the effects of ALS-inducing mutations on the populations of the thermodynamic states in apo SOD1 that might be candidates for aggregation.

To this end, perturbations of the folding free energy landscapes of a set of five ALS-variants of SOD1 with survival times of less than 3 years [26], [27] were mapped; the wild-type-like variants [28], A4V, L38V, G93A and L106V, and the metal-binding variant, S134N. As shown in Figure 1, Ala4 is located in β1, adjacent to the subunit interface, and the side chain points towards the interior of the β-barrel. The side chains of Leu38 and Leu106 serve as “plugs” at the ends of the β-barrel and are located in the Greek loops [2] that connect β3 and β4 (loop II) and β6 and β7 (loop VI), respectively. Gly93 is located in loop IV that connects β5 and β6, and it is proximal to Leu38. Along with Leu38, Gly93 takes part in an extensive hydrogen-bond network between loop V and loop VI that is thought to stabilize the β-barrel [8], [29]. Ser134 participates in a hydrogen-bonding network that bridges the electrostatic and Zn binding loops and is largely exposed to solvent [8]. All of these variants retain the dimeric quaternary structure of WT-SOD1 and, with the exception of S134N, effectively bind copper and zinc ions. S134N is only partially-metallated when isolated from insect cells [30] or yeast [31].

This present paper reports the population analysis for the disulfide-containing apo-forms of these variants because they provide a common reference state for both wild-type-like and metal-binding variants and because apo-SOD1 is thought to be involved in the formation of aggregates [16], [32], [33]. The results provide the framework for a comprehensive quantitative analysis of the effects of ALS mutations on the populations of monomeric forms of SOD1 that may be responsible for aggregation.

## Results

### Mutagenesis

The A4V, L38V, G93A, L106V and S134N mutations were introduced into the AS-SOD1 background [21], denoted herein as WT, which contains the C6A/C111S mutations to eliminate irreversible unfolding reactions caused by disulfide interchange with the intra-subunit C57–C146 disulfide bond in the unfolded state or by spontaneous oxidation of the cysteines by molecular oxygen. The structure and the stability of disulfide-oxidized AS-SOD1 are very similar to those for wild-type SOD1 [22], [34].

### Overall Strategy

The relative populations of the dimeric native state, N_2_, and the monomeric states, M and U, for the ALS-variants were determined by performing a comprehensive equilibrium and kinetic analysis of their reversible urea-induced unfolding/refolding reactions. The resultant perturbations of the maxima and minima on the folding free energy surface, extrapolated to the absence of denaturant, and the Bolzmann equation provide the desired populations.

### Equilibrium Analysis

**Figure 2:**
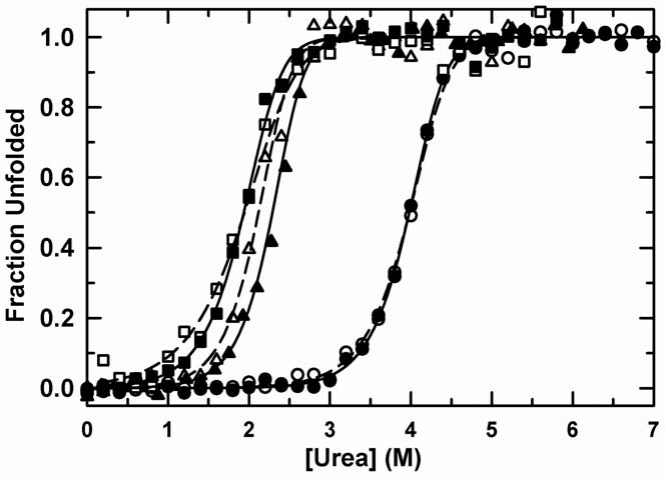
Normalized equilibrium unfolding transitions for SOD1 variants. Data were measured by far-UV CD at 230 nm at 10 µM monomer concentration. (•) WT, (□) A4V, (▴) L38V, (▵) G93A, (▪) L106V, (○) S134N. Solid (for open symbols) and dashed (for closed symbols) lines represent global fits of two to three protein concentrations to a two-state model. The corresponding free energies are listed in Table 1.

To ascertain the effect of the five disease-causing mutations on structure and thermodynamic stability, the equilibrium folding properties of WT and the apo-forms of the five variants were monitored by far-UV circular dichroism (CD) spectroscopy. The CD spectra are largely super-imposable (Figure S1), demonstrating that the secondary structures of these variants are not perturbed by the mutations. The mutations do, however, have a pronounced effect on the apparent thermodynamic stability of all but the S134N variant (Figure 2). The apparent stability was monitored by reversible urea-induced equilibrium unfolding transitions for all five variants at a series of different protein concentrations. As expected for the dissociation and unfolding of a dimeric protein, the midpoint of the unfolding transition, C*_m_*, shifts to higher denaturant concentration with increasing protein concentration (Figure S2). The destabilizing effect of the mutations can be seen by the dramatic shift in the midpoint of the transition C*_m_* from approximately 4 M urea for WT and S134N to below 2.5 M urea for A4V, L38V, G93A and L106V at 10 µM protein.

The perturbation in stability induced by the mutations was quantified by fitting the data to a 2-state dimer equilibrium unfolding model, 2U
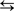
N_2_ (Figure 2, Figure S2 and Table 1) [21]. As expected from the nearly coincident titration curves in Figure 2, the stability, ΔG°, the denaturant dependence of the stability, the *m*-value, and the C*_m_* of S134N are within error of the values for WT. The apparent stabilities of the other variants, however, are significantly reduced: L38V and G93A are destabilized by 4 kcal mol^−1^ compared to WT, while A4V and L106V are further destabilized by approximately another 3 kcal mol^−1^ (Table 1). The *m*-values, which often correlate with the change in solvent accessible surface area accompanying unfolding [35], vary from ∼2.5 kcal mol^−1^ M^−1^ for the A4V variant to ∼4.1 kcal mol^−1^ M^−1^ for the G93A variant. A variation in the *m*-value of this magnitude is not expected for mutations that have little effect on the secondary structure (Figure S1) and retain the dimeric form. As will be shown below, a global kinetic analysis is required to obtain accurate estimates of the relative stabilities of the N_2_, M and U states for several of the SOD1 variants.

**Table 1:** Microscopic rate constants and kinetic *m* ^‡^-values obtained from the global kinetic analysis for WT SOD1 and the five ALS-inducing variants.^a^

	WT	A4V	L38V	G93A	L106V	S134N
**U 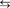 M**						
k*_f_*	(7.94±0.04)×10^−2^	(16.3±0.2)×10^−2^	(3.30±0.06)×10^−2^	(4.22±0.07)×10^−2^	(2.36±0.07)×10^−2^	(8.86±0.38)×10^−2^
*m^‡^_f_*	1.07±0.01	1.05±0.01	1.02±0.01	0.97±0.10	0.94±0.02	1.06±0.01
k*_u_*	0.20×10^−4^	12.0×10^−4^	3.0×10^−4^	20.0×10^−4^	6.00×10^−4^	0.12×10^−4^
*m^‡^_u_*	−0.59	−0.61	−0.80	−0.64	−0.67	−0.65
ΔG°_(U/M)_ b ^,^ c	−4.82±0.12	−2.86±0.05	−2.74±0.14	−1.78±0.18	−2.14±0.20	−5.18±0.13
*m*_(U/M)_ d	1.66±0.02	1.66±0.02	1.82±0.04	1.61±0.05	1.60±0.05	1.70±0.02
**2M 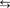 N_2_**						
k*_a_*	(0.86±0.05)×10^6^	(0.18±0.01)×10^6^	(0.12±0.02)×10^6^	(0.30±0.04)×10^6^	(0.073±0.016)×10^6^	(0.20±0.12)×10^6^
*m^‡^_a_*	0.38±0.01	0.38±0.11	0.46±0.03	0.60±0.03	0.54±0.05	0.36±0.07
k*_d_*	(7.45±0.27)×10^−4^	(223±12)×10^−4^	(5.57±0.07)×10^−4^	(1.78±0.02)×10^−4^	(15.1±0.6)×10^−4^	(2.22±0.19)×10^−4^
*m^‡^_d_*	−0.05±0.01	−0.08±0.01	−0.21±0.01	−0.26±0.01	−0.33±0.01	−0.13±0.01
ΔG°_(2M/N2)_ c	−12.2±0.1	−9.26±0.03	−11.2±0.1	−12.4±0.1	−10.3±0.13	−12.0±0.4
*m*_(2M/N2)_ d	0.42±0.01	0.46±0.11	0.68±0.03	0.86±0.03	0.87±0.05	0.50±0.07
*K_d_*	0.87 Nm	123 nM	4.6 nM	0.59 nM	21 nM	1.1 nM
**2U 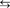 2M 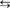 N_2_**						
ΔG°_(2U/N2)_ e	21.8±0.1	−15.0±0.1	−16.7±0.34	−15.9±0.4	−14.6±0.5	−22.4±0.6
*m*_(2U/N2)_ f	3.74±0.03	3.79±0.04	4.32±0.10	4.08±0.14	4.08±0.14	3.91±0.10
ΔG°(H_2_O)*_eq_*	−20.37±1.04	−11.58±0.62	−16.03±0.95	−15.79±0.73	−13.33±0.54	−19.72±0.40
*m_eq_*	3.39±0.24	2.53±0.26	4.07±0.39	4.11±0.30	3.42±0.25	3.17±0.10

a Units for the U
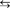
M reaction: k*_f_* and k*_u_*, s^−1^; *m^‡^_f_*, *m^‡^_u_* and *m*_(U/M)_, kcal (mol•monomer)^−1^ M^−1^; ΔG°_(U/M)_ kcal (mol•monomer)^−1^. Units for the 2M
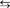
N_2_ reaction: k*_a_*, M^−1^ s^−1^; k*_d_*, s^−1^; *m^‡^_a_*, *m^‡^_d_*, and *m*_(2M/N2)_, kcal (mol•dimer)^−1^ M^−1^; ΔG°_(2M/N2)_ kcal (mol•dimer)^−1^. Units for the complete 2U
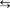
2M
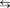
N_2_ reaction: ΔG°_(2U/N2)_ and ΔG°(H_2_O)*_eq_*, kcal (mol•dimer)^−1^; *m*_(2U/N2)_ and *m_eq_*, kcal (mol•dimer)^−1^ M^−1^.

b The errors represent fitting errors with the monomer unfolding rate held fixed. True errors are estimated to be closer to ±10%.

c Calculated according to ΔG°_(U/M)_ = −*RT*ln*K* = −*RT*ln(*k_f_*/*k_u_*) and ΔG°_(2M/N2)_ = −*RT*ln*K* = −*RT*ln(*k_a_*/*k_d_*).

d Calculated according to *m*_tot_ = |*m* ^‡^_u_|+|*m* ^‡^_f_| for the monomer and *m*_tot_ = |*m* ^‡^_d_|+|*m* ^‡^_a_| for the dimer.

e The total free energy of folding was calculated as ΔG°_(2U/N2)_ = 2 ΔG°_(U/M)_+ΔG°_(2M/N2)_.

f Calculated as *m*_(2U/N2)_ = 2(|*m^‡^_f_*|)+|*m^‡^_a_*|+2(|*m^‡^_u_*|)+|*m^‡^_d_*|.

### Kinetic Analysis

A more complete and accurate description of the perturbations of the maxima and minima on the folding free energy surfaces for the apo variants can be obtained by examination of the denaturant dependence of the refolding and unfolding kinetics (chevron analysis [36]). The advantage of this approach is that the perturbations of the global stability can be partitioned into the monomer folding step, U
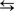
M, and the association step, 2M
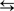
N_2_. This information is required to assess the effects of the mutations on the relative populations of the M and U states.

**Figure 3:**
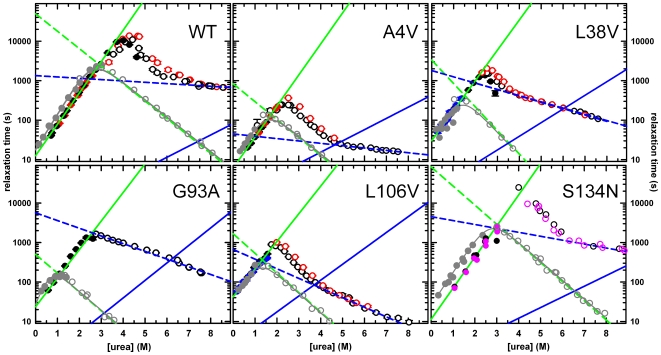
Observed refolding and unfolding relaxation times plotted as a function of final urea concentration. Refolding (filled circles) and unfolding (open circles) for WT, A4V, L38V, G93A, L106V, and S134N SOD1 were monitored by manual-mixing CD at 230 nm. The symbols correspond to the different final protein concentrations; 1–2 µM (pink), 4 µM (blue), 10 µM (black), and 30 µM (red). Lines are the inverse of the microscopic rate constants obtained in the global fits for the monomer refolding (solid green), monomer unfolding (dashed green), dimer association (solid blue), and dimer dissociation reaction (dashed blue). For clarity, the dimer association reaction has been normalized to 10 µM protein concentration. The relaxation times for the corresponding stable monomeric versions of these proteins are shown in grey circles, and the grey lines represent fits of the data to a two-state kinetic model, U
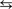
M.

The chevrons for the apo-ALS-variants have an overall urea dependence that is similar to that for apo-WT [21] (Figure 3), suggesting that the folding mechanism for the variants is consistent with the three-state folding mechanism observed for WT, 2U
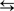
2M
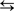
N_2_. The refolding relaxation times increase with increasing denaturant concentrations up to a maximum value near 2–2.5 M urea for A4V, L38V, G93A and L106V and near 4.0 M urea for S134N and WT. Above those urea concentrations, where unfolding is favored, the relaxation times decrease in a non-exponential fashion for WT and all variants except G93A. The relaxation times for WT and the A4V, L38V, L106V and S134N variants all roll-over to display a weak exponential dependence on the urea concentration under strongly unfolding conditions. The refolding relaxation times at 1 M urea vary by <3-fold from WT, while the unfolding relaxation times at 7 M urea vary ∼70-fold, from 13 s for L106V to 940 s for S134N.

The absence of a protein concentration dependence for refolding under any conditions for all of the variants is consistent with the rate-limiting unimolecular monomer folding reaction observed for SOD1 [9], [20], [21]. A signature of this mechanism is the appearance of protein concentration dependence in the unfolding relaxation times in the transition zone from 2–6 M urea. All of the variants show this behavior with the exception of the G93A variant (Figure 3). This effect has been observed previously [9], [20], [21] and reflects the increased apparent stability of the dimer at higher protein concentrations relative to the rate-limiting monomer unfolding transition state under these conditions. Under strongly unfolding conditions, >6 M urea, the exponential decrease in the relaxation time reflects the rate-limiting unimolecular dimer dissociation reaction [9], [20], [21].

Unlike for WT and all of the other variants, no change in the urea dependence of the rate-limiting step in unfolding was observed for G93A as the urea concentration was increased above 3 M. To test whether the unfolding, similar to WT, represents the disappearance of dimeric G93A, the time-resolved fluorescence (TR-FL) anisotropy decay of the intrinsic tryptophan was probed during unfolding. The rotational correlation time measurements demonstrated that the dimer, not the monomer, is present immediately after unfolding to 3.5 and 7.0 M urea for G93A (Figure S3 and Text S1). These results are consistent with the entire unfolding leg of the G93A chevron reflecting the dimer dissociation reaction, similar to the other variants and WT at high urea concentrations. As will be shown in the global analysis below, a faster monomer unfolding reaction and a slower dimer dissociation reaction for G93A lead to the uncoupling of these two reactions in the unfolding zone.

Determination of the folding reaction coordinate requires the rate constants for each kinetic step in the absence of denaturant. The rate constants for the first-order monomer folding reaction and the dimer dissociation reaction can be obtained to a reasonable degree of accuracy by linear extrapolation of the low and high urea segments of the chevron to 0 M urea and the relationship, *k* = 1/τ. However, these rate-limiting reactions in folding and unfolding preclude the extraction of the monomer unfolding rate constant and the association rate constant from the chevron plot. Hence, and as was done for WT [21], a global kinetic analysis of a comprehensive set of refolding and unfolding CD traces for each variant was performed to determine the microscopic rate constants for each step in the three-state folding mechanism.

### Global Analysis

In the global analysis, the kinetic traces at a series of urea concentrations ranging from 0.25 to 8.75 M urea and at several protein concentrations ranging from 1 to 40 µM for each variant were simultaneously fit to the three-state model, 2U
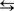
2M
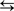
N_2_. To obtain reliable and robust parameters for the kinetic mechanism, it was necessary to fix the monomer unfolding rate constant, *k_u_* and its associated *m^‡^*-value to those obtained from stable monomeric variants of apo-AS-SOD1 (Figure 3). Other than this modification, a detailed description of the fitting procedure can be found elsewhere [21]. Kinetic traces with fits obtained from the global analysis applied to each of the mutants are shown in Figure S4.

The inverse of the microscopic rate constants predicted from the fits as a function of the urea concentration for the variants are projected on the chevrons in Figure 3, and the fit parameters are presented in Table 1. As expected, the predicted relaxation times for the monomer folding reaction, U→M, at low denaturant concentrations and the dimer dissociation reaction, N_2_→2M, at high denaturant concentrations for these rate-limiting reactions are in excellent agreement with the relaxation times extrapolated from low and high urea concentrations, respectively. The global analysis also reveals the predicted relaxation time for the monomer-monomer association reaction, 2M→N_2_, across the folding reaction coordinate from 0 to 8 M urea. The microscopic rate constant for the monomer unfolding reactions in the absence of urea, *k_u_*, extracted from the stable monomer variants varies 100-fold, ranging from 2.0×10^−5^ s^−1^ for WT to 2.0×10^−3^ s^−1^ for G93A (Figure 3 and Table 1). The association rate constants, *k_a_*, vary ∼12-fold, from 7.3×10^4^ M^−1^ s^−1^ for the L106V variant to 8.6×10^5^ M^−1^ s^−1^ for WT (Table 1).

It should be noted that the monomer unfolding rate constant, *k_u_*, and the monomer association rate constant, *k_a_*, for WT shown in Table 1 differ from the values previously reported using the global analysis procedure [21]. In that prior analysis, *k_u_* and *k_a_* were reported to be 9.45×10^−4^ s^−1^ and 2.0×10^9^ M^−1^s^−1^, respectively. The discrepancy reflects the existence of two minima on the chi-square surface for the WT global fit; the second minimum was only discovered by a reexamination motivated by the studies on the variants, which exhibited a considerably reduced association rate constant. The selection of the minimum with the significantly reduced *k_a_* for WT was further confirmed by complementary Förster resonance energy transfer studies directly probing the association reaction (J.A.Z., O.B. and C.R.M., unpublished data).

The minimum reported in the present study also exhibits a monomer unfolding rate for WT, A4V and L38V that is very similar to that found for the stable monomer construct. Although the monomer unfolding rate in the dimer context could not be determined to the same degree of confidence for the remaining variants by global analysis, the positive results for WT, A4V and L38V suggest that the chevron of the stable monomer construct is a good approximation of the monomer chevron in the dimer. Therefore, for consistency, in the present study the monomer unfolding parameters were fixed to those experimentally measured for the stable monomer variants, as described above. A similar approach has previously been used by Oliveberg and his colleagues in their studies on the folding of dimeric apo-AA-SOD1 [9], [20]. Although the presence of additional minima can never be completely ruled out, a random sampling of the parameter space consisting of at least 300 global fits was performed for WT and all variants to minimize this possibility and give added confidence that the parameters reported correspond to the global minimum (Figure S5).

**Figure 4:**
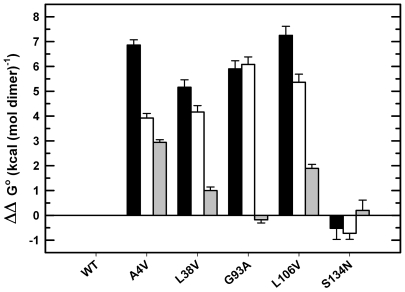
Perturbations in free energies for the ALS-variants. Perturbations relative to WT SOD1 were calculated from the parameters obtained in the global kinetic analysis for each protein. The black bars indicate the total change in free energy (2U
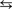
N_2_); the white bars indicate the change in free energy for the monomer (2U
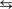
2M), and the grey bars indicate the change in free energy for the dimer association reaction (2M
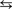
N_2_).

For each variant, these rate constants and their associated *m^‡^*-values can be used to calculate the free energies of the M state and the N_2_ state relative to the U state across the entire range of urea concentrations, recognizing that K_ij_ = *k_ij_/k_ji_* and ΔG°_ij_ = −RT lnK_ij_. With the exception of the wild-type-like stability of the S134N variant, ΔG° = −22.4 kcal mol^−1^, the standard state stabilities of the N_2_ state relative to the U state for the remaining variants are decreased by 6.8, 5.1, 5.9 and 7.2 kcal mol^−1^ for the A4V, L38V, G93A and L106V variants, respectively, relative to the value for WT of 21.8 kcal mol^−1^ (Table 1). These decreases in stability are partitioned between the free energy changes for the 2U
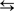
2M and the 2M
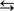
N_2_ steps in the three-state kinetic mechanism (Figure 4). The stabilities of the M states for A4V, L38V, G93A and L106V are decreased by 2.0, 2.1, 3.0 and 2.7 kcal mol^−1^, respectively. With the exception of the small increase of 0.2 kcal mol^−1^ for the G93A variant, the reduction in the free energy changes for the 2M
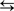
N_2_ step for A4V, L38V and L106V are 2.9, 1.0 and 1.9 kcal mol^−1^, respectively. The predicted K_d_ values for the dissociation of the dimer vary 200-fold, ranging from 0.6 nM for G93A to 123 nM for A4V (Table 1).

**Figure 5:**
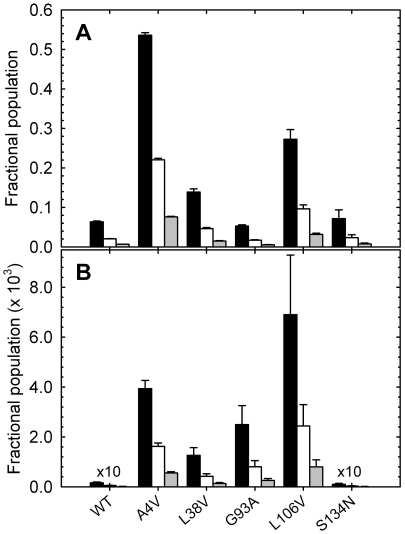
Fractional populations of monomeric species. The fractional populations of (A) the monomer and (B) the unfolded state at equilibrium and 0 M urea for 0.1 µM (black), 1 µM (white) and 10 µM (grey) protein concentration were calculated based on the parameters from the global kinetic analysis (Table 1). The populations of the unfolded states for WT SOD1 and S134N are multiplied by 10 in panel B to make them visible.

The free energy changes between states in the absence of urea, along with their associated *m*-values, can be used to estimate the relative populations of the N_2_, M and U states at equilibrium [21]. Potentially relevant to the misfolding/aggregation hypothesis for toxicity in ALS, the relative populations of the M state for A4V (7.6%), L38V (1.5%) and L106V (3.2%), increase by 2 to 12-fold compared to WT (0.65%) at 10 µM protein concentration and in the absence of denaturant (Figure 5A). However, the relative population of the M states for G93A (0.54%) and S134N (0.74%) are comparable to WT. By contrast, the U state shows a significant enhancement in population for all of the ALS variants compared to WT, with the exception of S134N. Although the relative populations of the unfolded, U, state for A4V, L38V, G93A and L106V are less than 0.1% at 10 µM protein, the populations only differ by 6-fold from each other and are 80 to 480-fold higher than for WT (Figure 5B). The population of U for S134N is comparable to WT.

**Figure 6:**
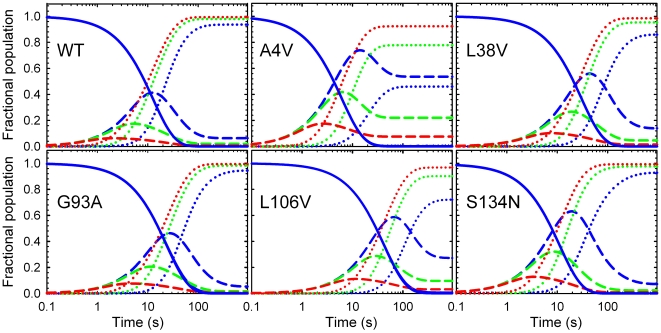
Kinetic species plots. Kinetic refolding species plots for U (solid line), M (dashed line) and N_2_ (dotted lines) were derived from the parameters obtained in the global fit (Table 1). Protein concentrations are 0.1 µM (blue), 1 µM (green) and 10 µM (red).

In addition to their populations at equilibrium, the populations of N_2_, M and U can also be calculated as a function of refolding time. The kinetic species plots derived from the global analysis parameters in Table 1 are shown in Figure 6 for each of the variants. Although modest enhancements of M are observed for A4V, L38V and L106V compared to WT, the most striking feature of these kinetic species plots is the remarkably long lifetime of the U state (∼10 s) for all of the variants. The U state of L38V, G93A and L106V persists for even longer times than for WT during refolding, while the lifetime of the U state is comparable for S134N and slightly shorter for A4V. A time-resolved small angle x-ray scattering study of WT showed that U undergoes very little compaction under folding conditions [21].

## Discussion

**Figure 7:**
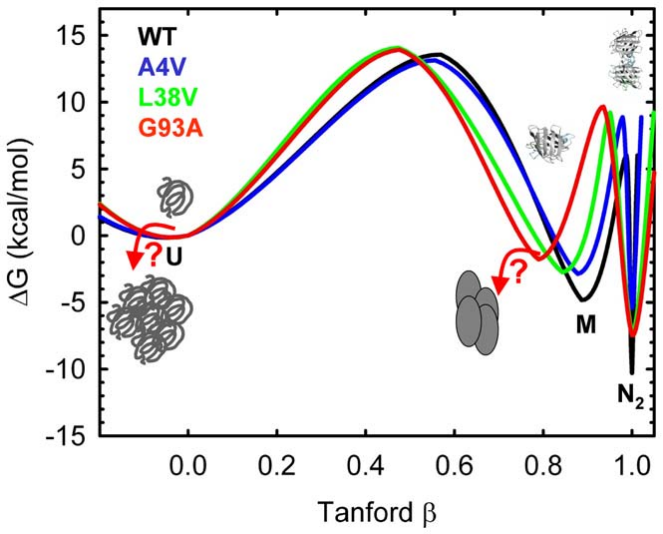
Effect of ALS-causing variants on the free energy landscape of SOD1. The folding reaction coordinates at 10 µM protein under native conditions are calculated from the global fits based on the results of *in vitro* kinetic measurements. The Tanford β value is used as the order parameter and is calculated from the kinetic *m*-values in Table 1 according to β = *m_f_/(m_f_−m_u_)*. This order parameter is expected to be proportional to solvent accessible surface area. The black line represents the folding reaction coordinate of the WT protein, which is nearly coincident with the reaction coordinate for the S134N variant (not shown). Other disease mutations result in selective destabilization of the dimer and/or significant destabilization of the monomer. The A4V variant (blue) predominately affects the dimer stability, whereas the G93A variant (red) has the largest effect on the monomer stability. The L38V (green) and L106V (not shown) variants affect both steps. Based on the enhanced populations of partially-folded forms observed for the ALS-causing variants, aggregation (red arrows) is hypothesized to proceed from the monomer (M) or unfolded (U) species. The wild-type like reaction coordinate observed for the metal-deficient S134N suggests that even WT SOD can aggregate in the absence of metals. Although the reaction coordinate shown is semi-quantitative, one caveat to including both monomers and dimers i a single reaction coordinate is that it is necessary to plot them with respect to different reference states. The part of the reaction coordinate corresponding to the monomer reaction is, therefore, scaled with respect to a monomer reference state and the bimolecular step is scaled with respect to a dimer reference state. A prefactor of 1×10^9^ s^−1^ and 1×10^9^ M^−1^s^−1^ was used for the unimolecular and bimolecular steps, respectively, in relating the rate to an activation free-energy using Kramers theory.

The global analysis of the kinetic folding data for apo-AS-SOD1 (WT) and five metal-free ALS-inducing variants, supported by the kinetic folding data for monomeric versions of these variants, has enabled the mapping of the perturbations of the maxima and minima on the folding free energy surfaces resulting from mutation. This strategy reveals the partitioning of the perturbations in stability between the 2U
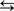
2M and the 2M
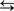
N_2_ steps in the three-state folding mechanism (Figure 4) and, thereby, the effects of the ALS-variants on the relative populations of these thermodynamic states at equilibrium (Figure 5) and during folding (Figure 6). The increase in the population of the folded or unfolded monomeric states is consistent with a role in initiating the oligomerization and/or aggregation reactions that might be responsible for toxicity in motor neurons (Figure 7).

The effects of ALS-causing variants on the free energy landscape of SOD1 shown in Figure 7 suggest a potential mechanism through which the ALS variants can oligomerize and exert toxicity by either a significant destabilization of the dimer, e.g., A4V, significant destabilization of the monomer, e.g., G93A, or a combination of both events, e.g., L38V and L106V. The decreased global stability for the A4V, L38V and L106V variants relative to WT is partitioned between the 2M
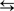
N_2_ step and the 2U
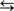
2M step in a way that leads to enhanced populations of folded monomers (Figure 5A). For these variants, the decreased association rate constants relative to WT suggest that the mutations have a substantial effect on the population of the association-competent fraction of the conformational ensemble representing the M state [37]. The data imply a bias towards a conformation of the monomer that may make it more likely to misfold and form small oligomers (Figure 7). Consistent with this idea, Akke and coworkers have recently used nuclear spin relaxation dispersion experiments to identify a short-lived, weakly populated monomeric conformation that could trigger oligomerization of monomeric apo-AA-SOD1 [38].

The similar decreases in stability of the dimeric and monomeric forms of G93A result in no change in the folded monomer population compared to WT; however, G93A, as well as A4V, L38V, and L106V show marked enhancement of the U state compared to WT (Figure 5B). Although the fractions of the unfolded states are small at equilibrium, <0.1%, the dramatic increase in the concentration, ∼200-fold, would be magnified by the order of the likely nucleation reaction, i.e., squared for second-order, cubed for third-order, etc., suggesting that amorphous aggregation of the U state may also play a role in toxicity (Figure 7). Consistent with this observation, Marklund and coworkers have shown that even small populations of misfolded mutant SOD1 can cause ALS [39]. Additionally, the long lifetime of the U state (10's of seconds) during folding (Figure 6) could potentially enhance the opportunities for U to participate in aberrant interactions.

The destabilizing effect of the mutations is not only limited to the apo-dimer. Crow and co-workers have shown that A4V and L38V, among other variants, destabilize metal binding as well [40]. Any other interference with metal loading, such as oxidative damage of SOD1 by superoxide, impaired zinc or copper homeostasis and diminished copper loading by its chaperone, would shift the equilibrium for SOD1 towards monomeric species [6], [7], [24]. For example, the co-expression of WT human SOD1 in mice carrying the A4V transgene exacerbates the toxicity of the A4V mutation rather than relieving it [41]. If the WT protein, which has a higher affinity for zinc than A4V [40], sequesters most of the available zinc, the A4V SOD1 might well be zinc deficient and more aggregation-prone than in the absence of the WT protein.

The toxicity of the S134N variant is intriguing in view of its wild-type like folding reaction coordinate (Table 1 and Figure 7) and population distribution (Figure 5). The results imply that for this metal-deficient variant [42], [43] and for metal-free WT SOD1, even small populations of the M and U states may be sufficient to cause aggregation and induce toxicity. Consistent with this prediction are the results of a recent study in which disulfide-reduced or unfolded WT SOD1 was found to be capable of initiating the aggregation of disulfide-oxidized apo-protein as well as zinc-bound protein [16].

A recent study by Prudencio *et al.* showed that ALS-inducing SOD1 variants with a short disease duration had a high propensity to aggregate in a cell culture model [44]. However, the aggregation propensity of the more than 30 ALS-variants studied did not correlate with any known biophysical property, including global stability, net charge, or enzymatic activity. The enhanced populations of unfolded monomeric forms for the ALS-variants of SOD1 may provide the key. The high aggregation propensities of A4V and G93A compared to the relatively low aggregation propensity of S134N are consistent with the perturbations in the population of unfolded monomeric forms observed in our study; unfortunately, L38V and L106V were not tested in the study by Prudencio *et al.* Although these aggregation-prone unfolded species would be a logical target for chaperone-assisted folding and/or proteosome degradation, the diminished potency of these homeostasis mechanisms over time [45] could ultimately lead afflicted neurons to succumb to cell death. Thus, the long-delayed onset of familial ALS, ∼45 years on average [26], [27], might be understood, in part, by the enhanced populations of monomeric species in ALS variants (Figure 7).

## Materials and Methods

### Protein Purification

All materials and methods employed have previously been described [21]. The ALS-inducing variants were introduced into the pseudoWT C6A/C111S background to enhance reversibility of folding [46], and the monomeric proteins also contained the F50E/G51E mutations required to prevent dimerization [47], [48]. Recombinant proteins were expressed in and purified from BL21-Gold(DE3) PLysS cells (Stratagene®, Inc. Cedar Creek, TX). When required, the protein was purified twice over an anion exchange QSepharose XL resin and/or by gel filtration using a Sephacryl 200HR column (GE Healthcare, Piscataway, NJ). Protein integrity was assessed by measuring the molecular weight with LC-ESI mass spectrometry. All experiments were performed using the apo form of the proteins, which were prepared as described previously [21]. The protein concentrations were calculated using an extinction coefficient of 10,800 M^−1^ cm^−1^ for the dimeric variants [49] and 5,400 M^−1^ cm^−1^ for the monomeric variants [21], and all protein concentrations are given in monomer units. The standard buffer used in all experiments was 10 mM potassium phosphate, 1 mM K_2_EDTA, pH 7.2, and the temperature was 20°C.

### Equilibrium and Kinetic Measurements

All equilibrium and kinetic measurements were performed on a Jasco-810 spectropolarimeter as described previously [21]. For the manual-mixing kinetic measurements, the signal change upon refolding and unfolding was monitored at 230 nm, and the mixing ratios varied from 1∶3 to 1∶28 depending on the final protein and urea concentrations, which ranged from 1–30 µM protein and 0.4–7 M urea, respectively. All equilibrium and kinetic measurements were analyzed according to published methods [21].

### Global Analysis Methods

The raw unfolding and refolding kinetic traces at varying protein and urea concentrations were globally fit to a 3-state kinetic mechanism, N_2_ 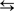
2M
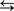
2U, using a Levenberg-Marquardt non-linear least squares fitting algorithm in the in-house fitting package Savuka, as described previously [21], [50]. The concentrations of all of the species were obtained by numerical solution of the coupled kinetic rate equations given below using a Runge-Kutta algorithm with an adaptive step size:
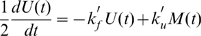


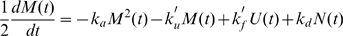


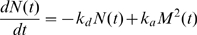
where *U(t)*, *M(t)* and *N(t)* are the concentrations of the unfolded state, folded monomeric state and dimeric native state at time *t*, respectively. The rates *k'_f_*, *k'_u_*, *k_a_* and *k_d_* are the monomer folding, unfolding, association and dimer dissociation rates, respectively. The rates *k'*_f_ and *k'_u_* for the monomer folding reaction above (2U
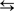
2M) are related to those reported in Table 1 for the U
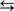
M folding reaction by multiplying by 2, i.e., *k*_f_ = 2*k'*_f_ and *k*_u_ = 2*k'_u_*. The rate constant at any given urea concentration, *k_xy_*, was expressed in terms of the rate in the absence of urea, *k°_xy_*, and the *m*- value:
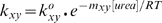
The equilibrium free-energy at standard state conditions, i.e. in the absence of urea and 1 M reactants and products, between species *x* and *y* is obtained as:
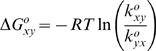
This gives the free-energy in the monomer reference state for the U to M folding reaction and the free-energy in the dimer reference state for the 2M to N_2_ folding reaction. The free-energy for the U to M folding reaction needs to be multiplied by a factor of 2 to obtain the free-energy in the dimer reference state:
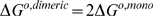
Because free-energy is an extensive property, the free energy is reported in the dimer reference state. For computational efficiency, kinetic traces comprising ∼1000 points were logarithmically averaged to yield ∼100 points evenly spaced in log-time and then fit simultaneously using an iterative procedure as previously described [21], [50]. Adjustable global, i.e. linked, parameters in the optimization consisted of the microscopic rate constants, *k^o^_xy_*, the kinetic *m* values, m*_xy_*, the Z-values, a normalized measure of the extent to which the intermediate resembles the unfolded state, 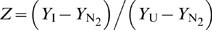, and the native and unfolded state baselines. The Z-values for all of the variants are close to zero, consistent with the similar CD spectra for the monomeric and dimeric variants [51]. The N_2_ and M species are discriminated kinetically by the change in the nature of the rate-limiting step in the transition region. The protein concentration was allowed to vary by up to 10% to account for the accuracy of the concentration measurement by absorbance.

Each optimization began by solving for the equilibrium concentration of all species under the starting conditions and then correcting for the dilution ratio. Although the starting conditions were, to a good approximation, fully native dimer or fully unfolded monomer, this approach was used to increase the generality of the kinetic model and to insure that the numerical algorithm could accommodate all starting conditions with equal accuracy. The initial concentrations were obtained from the following equations with the rates corresponding to the rates under the initial conditions instead of the final conditions:
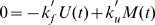


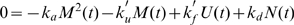


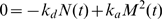
This iterative procedure was repeated until the kinetic parameters were optimized to yield the best fit. The goodness of fit was evaluated by the randomness of the residuals and the reduced-χ^2^.

## Supporting Information

Text S1 Time-resolved Fluorescence (TR-FL) Anisotropy Experimental Methods.
(0.03 MB DOC)

Figure S1 Circular dichroism spectra of apo-AS-SOD1 and the metal-free ALS-causing variants show well-folded global structures. Solid lines denote CD spectra at a protein concentration of 10 uM in 10 mM potassium phosphate, pH 7.2, 1 mM EDTA, 20 C: WT (black), A4V (red), L38V (green), G93A (pink), L106V (blue), and S134N (olive). The dashed line indicates the unfolded spectrum of WT in 7 M urea; the unfolded spectra of all of the ALS-causing variants are coincident with the WT spectrum.
(0.50 MB TIF)

Figure S2 Equilibrium unfolding traces monitored by CD at 230 nm for ALS variants. Protein concentrations are ((open circles) 4 uM, (closed circles) 10 uM, and (open squares) 30 uM monomer)) for (A) WT, (B) A4V, (C) L38V, (D) G93A, (E) L106V, and (F) S134N. The lines are from a global fit of 4–6 singular value decomposition vectors for each variants as described in Svensson et al. [1] (solid line, 4 uM; dashed line 10 uM; dotted line 30 uM).
(2.06 MB TIF)

Figure S3 Time-resolved fluorescence anisotropy of G93A. The colored lines depict the anisotropy decay at various unfolding timepoints in (A) 3.5 M and (B) 7 M urea. (A,B) light blue - 0 s, pink - 270 s, green - 570 s, yellow - 1170 s, and red - 1770 s. (C) The 0 s time point anisotropy decay for G93A at 3.5 M (light blue) and 7 M (blue) urea are compared to the equilibrium anisotropy decays of native (orange) and unfolded (3.5 M - hatched red line; 7 M - hatched black line) G93A and to those for native AS-SOD1 (black) and mAS-SOD1 (grey) [1]. The close agreement between the decays for dimeric WT and G93A SOD1 demonstrates that a dimer is the initial species in the unfolding reaction. The protein concentration was 10 uM monomer for all measurements.
(3.94 MB TIF)

Figure S4 Representative kinetic refolding and unfolding traces for WT and the five ALS-inducing SOD1 variants. The global fit using the model N2to2Mto2U is overlaid on the log-sampled data for each data set. The final urea concentration of each trace is color coded according to the color bar to the right of each panel. For WT, 29 unfolding traces at a protein concentration of 5 and 15 uM and 23 refolding traces at 5 and 15 uM are shown. For A4V, 16 representative refolding traces ranging from 4 to 20 uM protein concentration and 16 representative unfolding traces ranging from 2 to 10 uM protein concentration are shown. For L38V, 16 representative refolding traces at 10 uM protein concentration and 13 representative unfolding traces at 6 uM and 11.7 uM are shown. For G93A, 9 refolding traces at 10 uM final protein concentration and 14 representative unfolding traces spanning the 7.5 uM to 30 uM protein concentration range are shown. For L106V, 18 refolding traces at 3 and 9 uM protein concentration and 18 unfolding traces ranging from 4 to 30 8 uM protein concentration are shown. For S134N, 6 representative refolding traces at 10 uM protein concentration and 17 unfolding traces at 10 and 20 uM are shown.
(8.16 MB TIF)

Figure S5 Rigorous error analysis for the association rate, ka, for WT, S134N and G93A SOD1 variants. The WT chi-square error surface (top) is representative of a case where the association rate is well determined. The S134N variant (middle) displays the worst-case scenario, a situation where the upper bound on the association rate could not be determined with the same degree of confidence as for the wild-type protein. A typical case is represented by the G93A variant (bottom), where the upper and lower bounds given by the 68% confidence interval are well defined. The 68% confidence level was calculated based on an F-test. The error analysis was done by starting several hundred global fits from pseudo-random starting parameters with the association rate fixed to the value indicated by the x-coordinate of each point on the plot. The solid line outlining the minimum is drawn to aid the eye.
(0.99 MB TIF)

## References

[1] Rothstein JD (2009). Current hypotheses for the underlying biology of amyotrophic lateral sclerosis.. Ann Neurol.

[2] Getzoff ED, Tainer JA, Stempien MM, Bell GI, Hallewell RA (1989). Evolution of CuZn superoxide dismutase and the Greek key beta-barrel structural motif.. Proteins.

[3] Perry JJ, Shin DS, Getzoff ED, Tainer JA (2010). The structural biochemistry of the superoxide dismutases.. Biochim Biophys Acta.

[4] McCord JM, Fridovich I (1969). Superoxide dismutase. An enzymic function for erythrocuprein (hemocuprein).. J Biol Chem.

[5] Valentine JS, Hart PJ (2003). Misfolded CuZnSOD and amyotrophic lateral sclerosis.. Proc Natl Acad Sci U S A.

[6] Kayatekin C, Zitzewitz JA, Matthews CR (2008). Zinc binding modulates the entire folding free energy surface of human Cu,Zn superoxide dismutase.. J Mol Biol.

[7] Rumfeldt JA, Lepock JR, Meiering EM (2009). Unfolding and folding kinetics of amyotrophic lateral sclerosis-associated mutant Cu,Zn superoxide dismutases.. J Mol Biol.

[8] Tainer JA, Getzoff ED, Beem KM, Richardson JS, Richardson DC (1982). Determination and analysis of the 2 A-structure of copper, zinc superoxide dismutase.. J Mol Biol.

[9] Lindberg MJ, Normark J, Holmgren A, Oliveberg M (2004). Folding of human superoxide dismutase: disulfide reduction prevents dimerization and produces marginally stable monomers.. Proc Natl Acad Sci U S A.

[10] Hornberg A, Logan DT, Marklund SL, Oliveberg M (2007). The coupling between disulphide status, metallation and dimer interface strength in Cu/Zn superoxide dismutase.. J Mol Biol.

[11] Bruijn LI, Houseweart MK, Kato S, Anderson KL, Anderson SD (1998). Aggregation and motor neuron toxicity of an ALS-linked SOD1 mutant independent from wild-type SOD1.. Science.

[12] Cleveland DW, Rothstein JD (2001). From Charcot to Lou Gehrig: deciphering selective motor neuron death in ALS.. Nat Rev Neurosci.

[13] Bruijn LI, Miller TM, Cleveland DW (2004). Unraveling the mechanisms involved in motor neuron degeneration in ALS.. Annu Rev Neurosci.

[14] Rakhit R, Robertson J, Vande Velde C, Horne P, Ruth DM (2007). An immunological epitope selective for pathological monomer-misfolded SOD1 in ALS.. Nat Med.

[15] Wang J, Farr GW, Zeiss CJ, Rodriguez-Gil DJ, Wilson JH (2009). Progressive aggregation despite chaperone associations of a mutant SOD1-YFP in transgenic mice that develop ALS.. Proc Natl Acad Sci U S A.

[16] Chattopadhyay M, Durazo A, Sohn SH, Strong CD, Gralla EB (2008). Initiation and elongation in fibrillation of ALS-linked superoxide dismutase.. Proc Natl Acad Sci USA.

[17] Jonsson PA, Graffmo KS, Andersen PM, Brannstrom T, Lindberg M (2006). Disulphide-reduced superoxide dismutase-1 in CNS of transgenic amyotrophic lateral sclerosis models.. Brain.

[18] Lindberg MJ, Tibell L, Oliveberg M (2002). Common denominator of Cu/Zn superoxide dismutase mutants associated with amyotrophic lateral sclerosis: decreased stability of the apo state.. Proc Natl Acad Sci U S A.

[19] Stathopulos PB, Rumfeldt JA, Scholz GA, Irani RA, Frey HE (2003). Cu/Zn superoxide dismutase mutants associated with amyotrophic lateral sclerosis show enhanced formation of aggregates in vitro.. Proc Natl Acad Sci U S A.

[20] Lindberg MJ, Bystrom R, Boknas N, Andersen PM, Oliveberg M (2005). Systematically perturbed folding patterns of amyotrophic lateral sclerosis (ALS)-associated SOD1 mutants.. Proc Natl Acad Sci U S A.

[21] Svensson AK, Bilsel O, Kondrashkina E, Zitzewitz JA, Matthews CR (2006). Mapping the folding free energy surface for metal-free human Cu,Zn superoxide dismutase.. J Mol Biol.

[22] Vassall KA, Stathopulos PB, Rumfeldt JA, Lepock JR, Meiering EM (2006). Equilibrium thermodynamic analysis of amyotrophic lateral sclerosis-associated mutant apo Cu,Zn superoxide dismutases.. Biochemistry.

[23] Rumfeldt JA, Stathopulos PB, Chakrabarrty A, Lepock JR, Meiering EM (2006). Mechanism and thermodynamics of guanidinium chloride-induced denaturation of ALS-associated mutant Cu,Zn superoxide dismutases.. J Mol Biol.

[24] Mulligan VK, Kerman A, Ho S, Chakrabartty A (2008). Denaturational stress induces formation of zinc-deficient monomers of Cu,Zn superoxide dismutase: implications for pathogenesis in amyotrophic lateral sclerosis.. J Mol Biol.

[25] Nordlund A, Leinartaite L, Saraboji K, Aisenbrey C, Grobner G (2009). Functional features cause misfolding of the ALS-provoking enzyme SOD1.. Proc Natl Acad Sci U S A.

[26] Cudkowicz ME, McKenna-Yasek D, Sapp PE, Chin W, Geller B (1997). Epidemiology of mutations in superoxide dismutase in amyotrophic lateral sclerosis.. Ann Neurol.

[27] Wang Q, Johnson JL, Agar NY, Agar JN (2008). Protein aggregation and protein instability govern familial amyotrophic lateral sclerosis patient survival.. PLoS Biol.

[28] Valentine JS, Doucette PA, Zittin Potter S (2005). Copper-zinc superoxide dismutase and amyotrophic lateral sclerosis.. Annu Rev Biochem.

[29] Shipp EL, Cantini F, Bertini I, Valentine JS, Banci L (2003). Dynamic properties of the G93A mutant of copper-zinc superoxide dismutase as detected by NMR spectroscopy: implications for the pathology of familial amyotrophic lateral sclerosis.. Biochemistry.

[30] Tiwari A, Xu Z, Hayward LJ (2005). Aberrantly increased hydrophobicity shared by mutants of Cu,Zn-superoxide dismutase in familial amyotrophic lateral sclerosis.. J Biol Chem.

[31] Elam JS, Taylor AB, Strange R, Antonyuk S, Doucette PA (2003). Amyloid-like filaments and water-filled nanotubes formed by SOD1 mutant proteins linked to familial ALS.. Nat Struct Biol.

[32] Banci L, Bertini I, Durazo A, Girotto S, Gralla EB (2007). Metal-free superoxide dismutase forms soluble oligomers under physiological conditions: a possible general mechanism for familial ALS.. Proc Natl Acad Sci U S A.

[33] Furukawa Y, Kaneko K, Yamanaka K, O'Halloran TV, Nukina N (2008). Complete loss of post-translational modifications triggers fibrillar aggregation of SOD1 in the familial form of amyotrophic lateral sclerosis.. J Biol Chem.

[34] Hallewell RA, Imlay KC, Lee P, Fong NM, Gallegos C (1991). Thermostabilization of recombinant human and bovine CuZn superoxide dismutases by replacement of free cysteines.. Biochem Biophys Res Commun.

[35] Myers JK, Pace CN, Scholtz JM (1995). Denaturant m values and heat capacity changes: relation to changes in accessible surface areas of protein unfolding.. Protein Sci.

[36] Matthews CR (1987). Effect of point mutations on the folding of globular proteins.. Methods Enzymol.

[37] Zitzewitz JA, Ibarra-Molero B, Fishel DR, Terry KL, Matthews CR (2000). Preformed secondary structure drives the association reaction of GCN4-p1, a model coiled-coil system.. J Mol Biol.

[38] Teilum K, Smith MH, Schulz E, Christensen LC, Solomentsev G (2009). Transient structural distortion of metal-free Cu/Zn superoxide dismutase triggers aberrant oligomerization.. Proc Natl Acad Sci U S A.

[39] Jonsson PA, Ernhill K, Andersen PM, Bergemalm D, Brannstrom T (2004). Minute quantities of misfolded mutant superoxide dismutase-1 cause amyotrophic lateral sclerosis.. Brain.

[40] Crow JP, Sampson JB, Zhuang Y, Thompson JA, Beckman JS (1997). Decreased zinc affinity of amyotrophic lateral sclerosis-associated superoxide dismutase mutants leads to enhanced catalysis of tyrosine nitration by peroxynitrite.. J Neurochem.

[41] Deng HX, Shi Y, Furukawa Y, Zhai H, Fu R (2006). Conversion to the amyotrophic lateral sclerosis phenotype is associated with intermolecular linked insoluble aggregates of SOD1 in mitochondria.. Proc Natl Acad Sci U S A.

[42] Hayward LJ, Rodriguez JA, Kim JW, Tiwari A, Goto JJ (2002). Decreased metallation and activity in subsets of mutant superoxide dismutases associated with familial amyotrophic lateral sclerosis.. J Biol Chem.

[43] Tiwari A, Hayward LJ (2003). Familial amyotrophic lateral sclerosis mutants of copper/zinc superoxide dismutase are susceptible to disulfide reduction.. J Biol Chem.

[44] Prudencio M, Hart PJ, Borchelt DR, Andersen PM (2009). Variation in aggregation propensities among ALS-associated variants of SOD1: Correlation to human disease.. Hum Mol Genet.

[45] Balch WE, Morimoto RI, Dillin A, Kelly JW (2008). Adapting proteostasis for disease intervention.. Science.

[46] Lepock JR, Frey HE, Hallewell RA (1990). Contribution of conformational stability and reversibility of unfolding to the increased thermostability of human and bovine superoxide dismutase mutated at free cysteines.. J Biol Chem.

[47] Banci L, Benedetto M, Bertini I, Del Conte R, Piccioli M (1998). Solution structure of reduced monomeric Q133M2 copper, zinc superoxide dismutase (SOD). Why is SOD a dimeric enzyme?. Biochemistry.

[48] Banci L, Bertini I, Cantini F, D'Onofrio M, Viezzoli MS (2002). Structure and dynamics of copper-free SOD: The protein before binding copper.. Protein Sci.

[49] Goto JJ, Gralla EB, Valentine JS, Cabelli DE (1998). Reactions of hydrogen peroxide with familial amyotrophic lateral sclerosis mutant human copper-zinc superoxide dismutases studied by pulse radiolysis.. J Biol Chem.

[50] Noel AF, Bilsel O, Kundu A, Wu Y, Zitzewitz JA (2009). The folding free-energy surface of HIV-1 protease: insights into the thermodynamic basis for resistance to inhibitors.. J Mol Biol.

[51] Kayatekin C, Zitzewitz JA, Matthews CR (2010). Disulfide-reduced ALS variants of copper zinc superoxide dismutase exhibit increased populations of unfolded species.. J Mol Biol in press.

[52] DeLano WL (2002). The PyMOL Molecular Graphics System.. http://www.pymol.org.

[53] Strange RW, Antonyuk SV, Hough MA, Doucette PA, Valentine JS (2006). Variable metallation of human superoxide dismutase: atomic resolution crystal structures of Cu-Zn, Zn-Zn and as-isolated wild-type enzymes.. J Mol Biol.

