# Supplementary material for: Metal-Free ALS Variants of Dimeric Human Cu,Zn-Superoxide Dismutase Have Enhanced Populations of Monomeric Species
Source: PLoS One. 2010 Apr 9;5(4):e10064. doi: 10.1371/journal.pone.0010064 (PMC2852398; doi:10.1371/journal.pone.0010064)
Supplement: Text S1 — Time-resolved Fluorescence (TR-FL) Anisotropy Experimental Methods. (0.03 MB DOC) [file pone.0010064.s001.doc]

**Text S1. Time-resolved Fluorescence (TR-FL) Anisotropy Experimental Methods.** The TR-FL anisotropy experiments for G93A were performed as previously described [1], with the exception that 128 histograms for each polarization were collected in 10 s or 30 s bins. The final protein concentration for all measurements was 10 μM in terms of monomer. TR-FL anisotropy was performed using the time-correlated single-photon counting method with the tripled-output (294 nm) of a diode-pumped titanium:sapphire laser (Coherent Inc., Palo Alto, CA) at a repetition rate of 3.8 MHz. Detection was in a T-format arrangement with Glan-Taylor polarizers in each path. The outputs from two fast photomultiplier tubes (Becker-Hickl, PMH100, Berlin, Germany) equipped with 350 nm bandpass filters (Semrock Inc., Rochester, NY) were fed into a router and time-correlated counting card (Becker-Hickl, SPC630). The G-factor was obtained from the integrated intensity of vertical and horizontal excited state decays using horizontally polarized excitation.

1. Svensson AK, Bilsel O, Kondrashkina E, Zitzewitz JA, Matthews CR (2006) Mapping the folding free energy surface for metal-free human Cu,Zn superoxide dismutase. J Mol Biol 364: 1084-1102.
