# Supplementary figures and images for: Metal-Free ALS Variants of Dimeric Human Cu,Zn-Superoxide Dismutase Have Enhanced Populations of Monomeric Species
Source: PLoS One. 2010 Apr 9;5(4):e10064. doi: 10.1371/journal.pone.0010064 (PMC2852398; doi:10.1371/journal.pone.0010064)

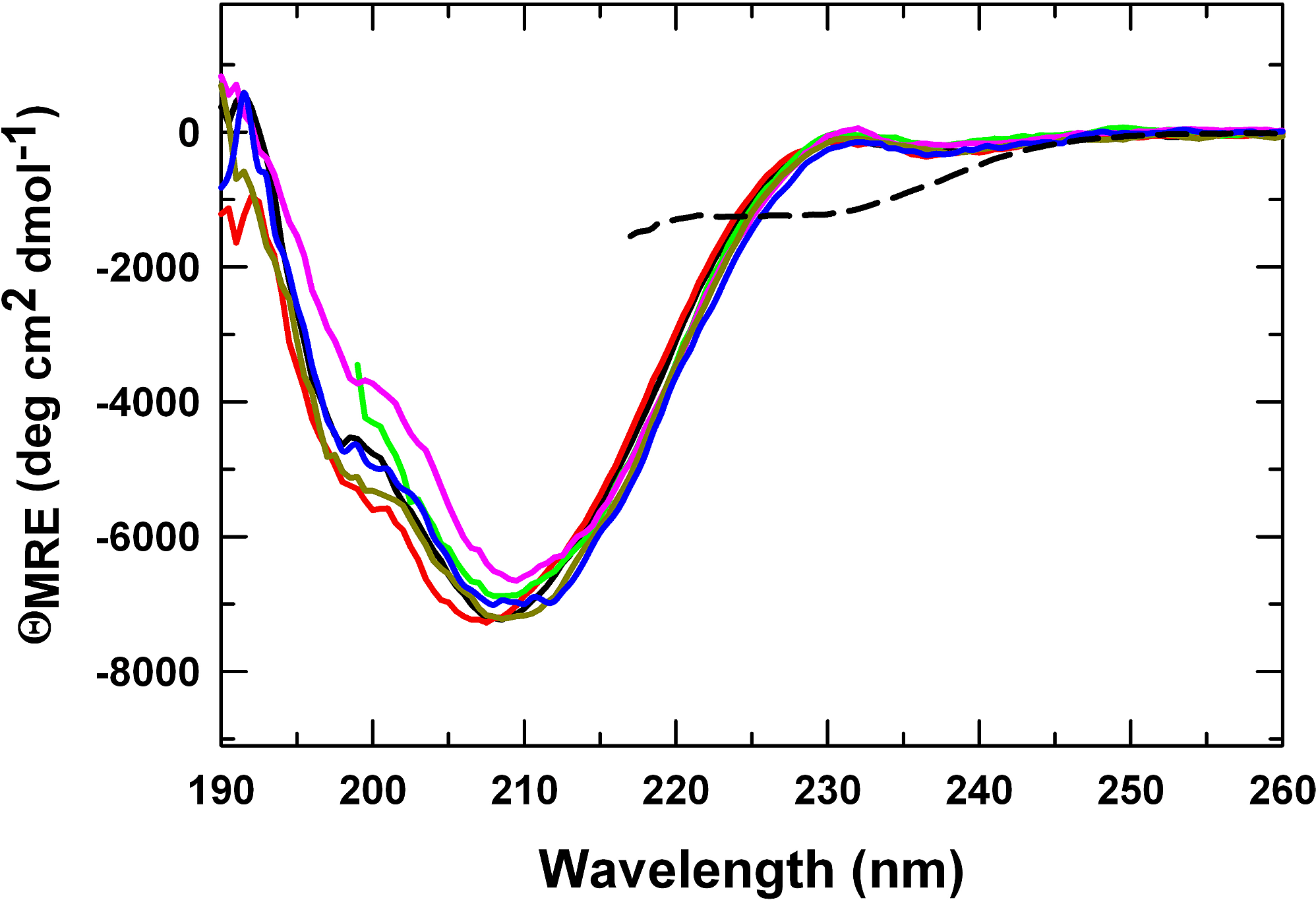

Supplement: Figure S1 — Circular dichroism spectra of apo-AS-SOD1 and the metal-free ALS-causing variants show well-folded global structures. Solid lines denote CD spectra at a protein concentration of 10 uM in 10 mM potassium phosphate, pH 7.2, 1 mM EDTA, 20 C: WT (black), A4V (red), L38V (green), G93A (pink), L106V (blue), and S134N (olive). The dashed line indicates the unfolded spectrum of WT in 7 M urea; the unfolded spectra of all of the ALS-causing variants are coincident with the WT spectrum. (0.50 MB TIF) [file pone.0010064.s002.tif]

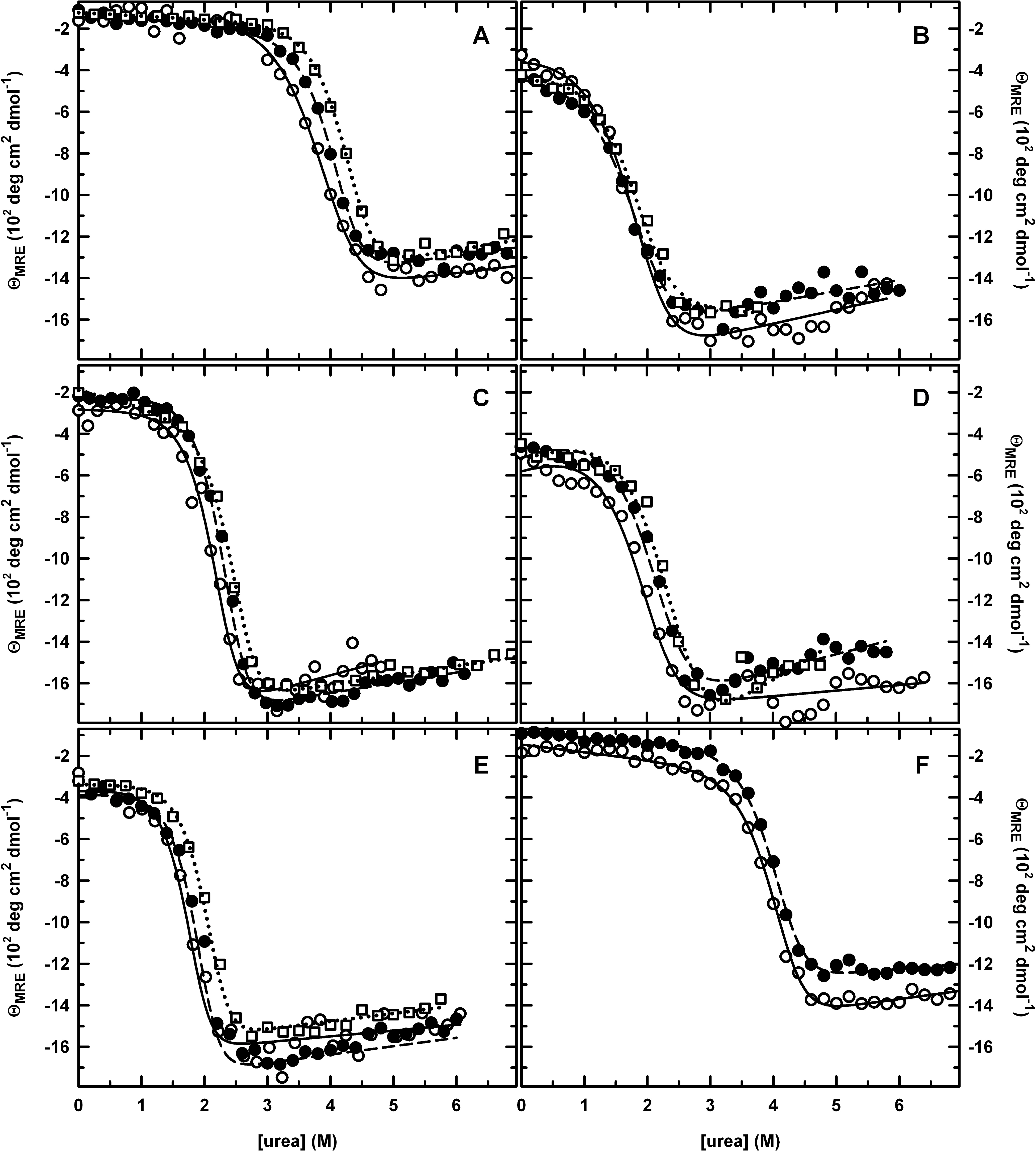

Supplement: Figure S2 — Equilibrium unfolding traces monitored by CD at 230 nm for ALS variants. Protein concentrations are ((open circles) 4 uM, (closed circles) 10 uM, and (open squares) 30 uM monomer)) for (A) WT, (B) A4V, (C) L38V, (D) G93A, (E) L106V, and (F) S134N. The lines are from a global fit of 4–6 singular value decomposition vectors for each variants as described in Svensson et al. [1] (solid line, 4 uM; dashed line 10 uM; dotted line 30 uM). (2.06 MB TIF) [file pone.0010064.s003.tif]

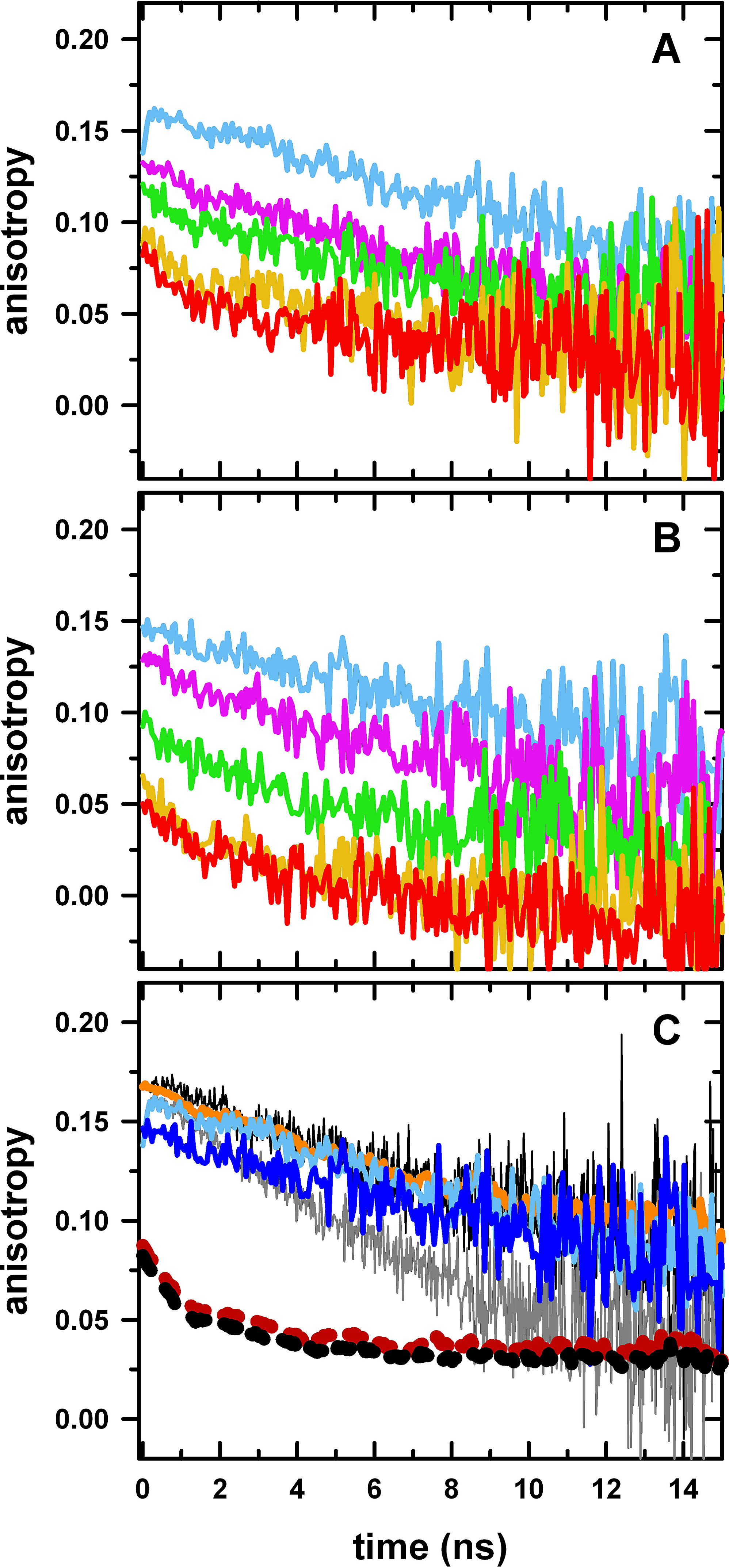

Supplement: Figure S3 — Time-resolved fluorescence anisotropy of G93A. The colored lines depict the anisotropy decay at various unfolding timepoints in (A) 3.5 M and (B) 7 M urea. (A,B) light blue - 0 s, pink - 270 s, green - 570 s, yellow - 1170 s, and red - 1770 s. (C) The 0 s time point anisotropy decay for G93A at 3.5 M (light blue) and 7 M (blue) urea are compared to the equilibrium anisotropy decays of native (orange) and unfolded (3.5 M - hatched red line; 7 M - hatched black line) G93A and to those for native AS-SOD1 (black) and mAS-SOD1 (grey) [1]. The close agreement between the decays for dimeric WT and G93A SOD1 demonstrates that a dimer is the initial species in the unfolding reaction. The protein concentration was 10 uM monomer for all measurements. (3.94 MB TIF) [file pone.0010064.s004.tif]

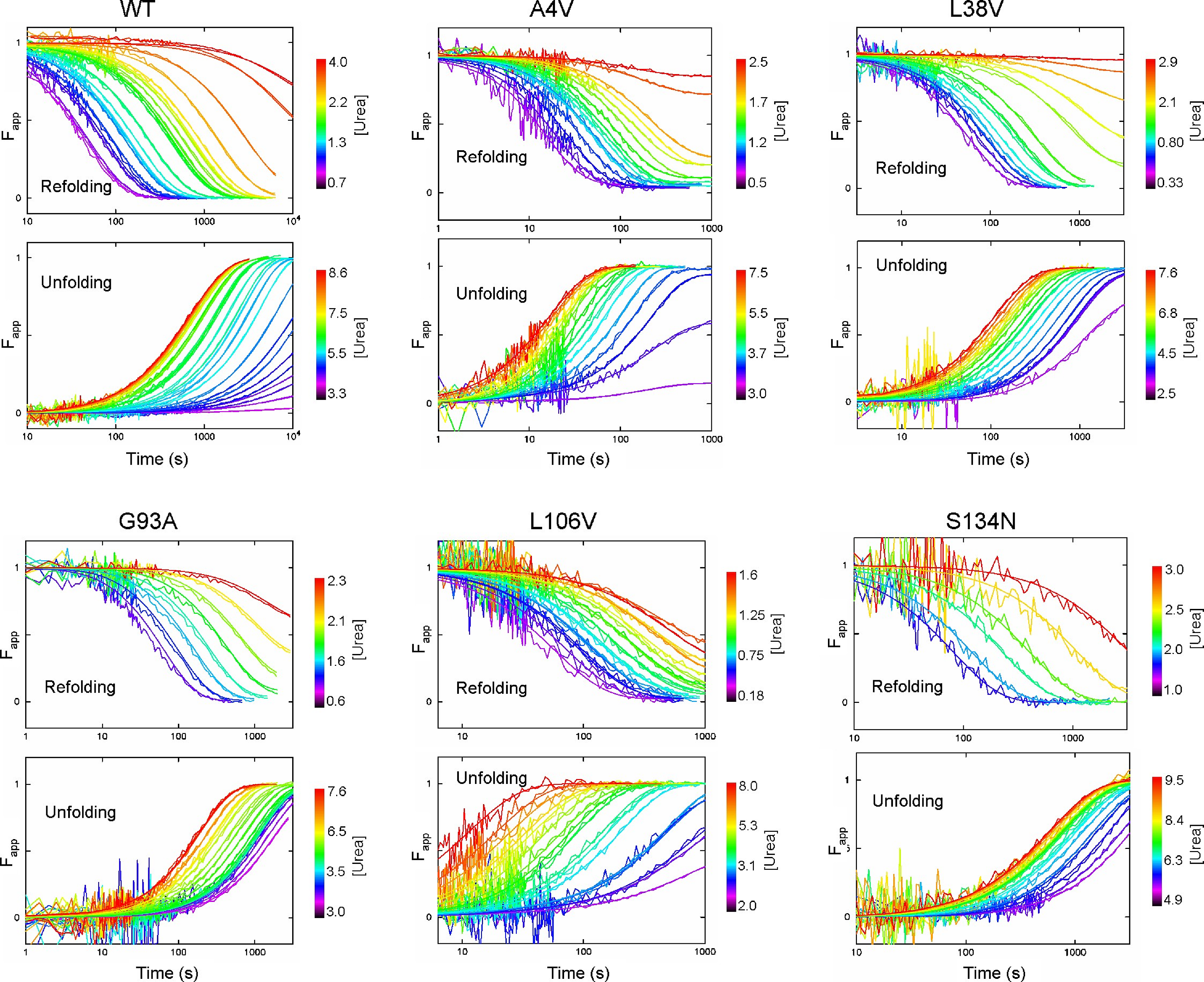

Supplement: Figure S4 — Representative kinetic refolding and unfolding traces for WT and the five ALS-inducing SOD1 variants. The global fit using the model N2to2Mto2U is overlaid on the log-sampled data for each data set. The final urea concentration of each trace is color coded according to the color bar to the right of each panel. For WT, 29 unfolding traces at a protein concentration of 5 and 15 uM and 23 refolding traces at 5 and 15 uM are shown. For A4V, 16 representative refolding traces ranging from 4 to 20 uM protein concentration and 16 representative unfolding traces ranging from 2 to 10 uM protein concentration are shown. For L38V, 16 representative refolding traces at 10 uM protein concentration and 13 representative unfolding traces at 6 uM and 11.7 uM are shown. For G93A, 9 refolding traces at 10 uM final protein concentration and 14 representative unfolding traces spanning the 7.5 uM to 30 uM protein concentration range are shown. For L106V, 18 refolding traces at 3 and 9 uM protein concentration and 18 unfolding traces ranging from 4 to 30 8 uM protein concentration are shown. For S134N, 6 representative refolding traces at 10 uM protein concentration and 17 unfolding traces at 10 and 20 uM are shown. (8.16 MB TIF) [file pone.0010064.s005.tif]

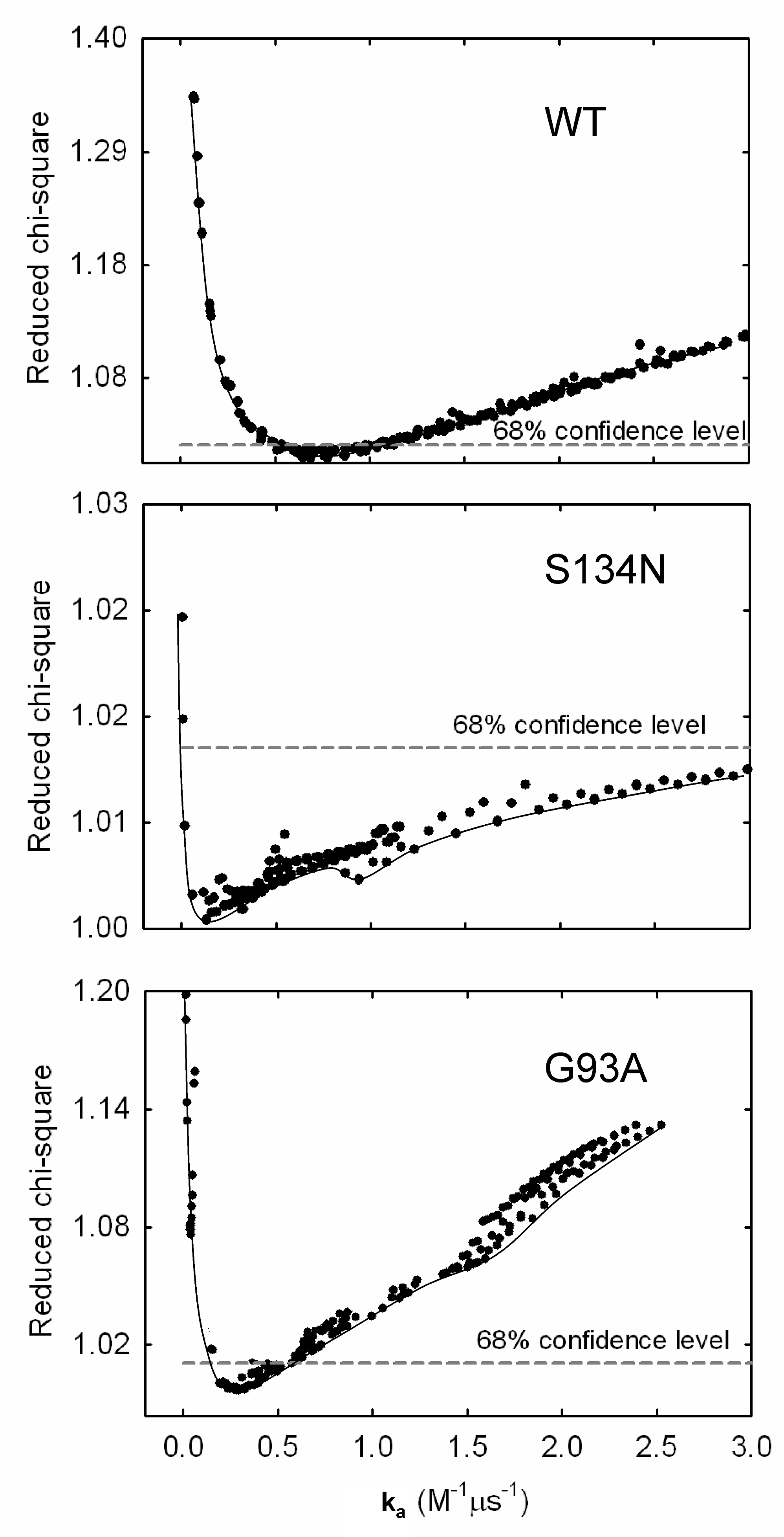

Supplement: Figure S5 — Rigorous error analysis for the association rate, ka, for WT, S134N and G93A SOD1 variants. The WT chi-square error surface (top) is representative of a case where the association rate is well determined. The S134N variant (middle) displays the worst-case scenario, a situation where the upper bound on the association rate could not be determined with the same degree of confidence as for the wild-type protein. A typical case is represented by the G93A variant (bottom), where the upper and lower bounds given by the 68% confidence interval are well defined. The 68% confidence level was calculated based on an F-test. The error analysis was done by starting several hundred global fits from pseudo-random starting parameters with the association rate fixed to the value indicated by the x-coordinate of each point on the plot. The solid line outlining the minimum is drawn to aid the eye. (0.99 MB TIF) [file pone.0010064.s006.tif]
